# Cervical cancer screening prevalence and its correlates in Cameroon: secondary data analysis of the 2018 demographic and health surveys

**DOI:** 10.1186/s12889-021-11024-z

**Published:** 2021-06-05

**Authors:** Joshua Okyere, Precious Adade Duodu, Livingstone Aduse-Poku, Pascal Agbadi, Jerry John Nutor

**Affiliations:** 1 Department of Population and Health, University of Cape Coast, Private Mail Bag, Cape Coast, Ghana; 2 Department of Nursing and Midwifery, School of Human and Health Sciences, University of Huddersfield, Queensgate, Huddersfield, England UK; 3 Department of Epidemiology, College of Public Health & Health Professions, College of Medicine, University of Florida, Gainesville, USA; 4 Department of Nursing, Faculty of Allied Health Sciences, College of Health Sciences, Kwame Nkrumah University of Science and Technology, Kumasi, Ghana; 5 Department of Family Health Care Nursing, School of Nursing, University of California San Francisco, San Francisco, CA USA

**Keywords:** Cervical Cancer, Screening, Cameroon, Firth-logit, Demographic and health surveys

## Abstract

**Background:**

Cervical cancer, although preventable, is the fourth most common cancer among women globally, and the second most common and deadliest gynaecological cancer in low-and-middle-income countries. Screening is key to the prevention and early detection of the disease for treatment. A few studies estimated the prevalence of cervical cancer screening and its correlates in Cameroon but relied on data that were limited to certain regions of the country. Therefore, this study sought to examine the prevalence and correlates of cervical cancer screening among Cameroonian women using current data that is nationally representative of reproductive-age women.

**Methods:**

We used secondary data from the 2018 Cameroon Demographic and Health Survey. Summary statistics were used for the sample description. We employed the Firth logistic regression using the “firthlogit” command in STATA-14 to perform the bivariate analyses between the outcome variable and each of the explanatory variables. Given that all the explanatory variables were statistically significant correlates, they were all adjusted for in a multivariable analysis. All analyses were performed in STATA version 14.

**Results:**

The proportion of Cameroonian women who have ever screened for cervical cancer continue to remain low at approximately 4%. In the adjusted model, women with the following sociodemographic characteristics have a higher likelihood of undergoing cervical cancer screening: ever undergone HIV screening (AOR = 4.446, 95% CI: 2.475, 7.986), being 24–34 years (AOR = 2.233, 95% CI: 1.606, 3.103) or 35–44 years (AOR = 4.008, 95% CI: 2.840, 5.657) or at least 45 years old (AOR = 5.895, 95% CI: 3.957, 8.784), having attained a post-secondary education (AOR = 1.849, 95% CI: 1.032, 3.315), currently (AOR = 1.551, 95% CI: 1.177, 2.043) or previously married (AOR = 1.572, 95% CI: 1.073, 2.302), dwelling in the richest household (AOR = 4.139, 95% CI: 1.769, 9.682), and residing in an urban area (AOR = 1.403, 95% CI: 1.004,1.960). Except for the North-West region, residing in some five regions, compared to Yaounde, was negatively associated with cervical cancer screening.

**Conclusion:**

Cervical cancer screening programs and policies should target Cameroonian women who are younger, less educated, and those in poor households and rural areas.

## Introduction

Cancers continue to be an increasing public health concern and remain the second leading cause of deaths globally [1]. In 2018, about 18.1 million new cases and 9.6 million cancer-related deaths occurred globally [2]. Cervical cancer, though preventable, remains the fourth most common cancer among women globally with an average age of diagnosis of 53 years [1, 3, 4]. Cervical cancer doubles as the second most common and deadliest gynaecological cancer in low-and-middle-income countries (LMICs) [3, 5]. In 2018, about 57,0000 new cases and an estimated 311,000 new deaths occurred globally, and these deaths are projected to increase by almost 50% by 2040 [3, 6]. The majority of the disease burden (about 85%) occur in Sub-Saharan Africa (SSA) [4, 7, 8], accounting for about 20.8% of all cancers in women and 14.2% of all cancer-related deaths in women [9]. Cervical cancer incidence in LMICs is almost twice as high and its death rates three times as high as those in high-income countries [10]. The human papillomavirus (HPV), an infection commonly acquired through sex, is the main cause of about 99% of cervical pre-cancer and cancer [6, 9]. It is noteworthy that HPV is closely associated with other preventable sexually transmitted infections including human immunodeficiency virus (HIV) [4, 11], with studies drawing a link between HIV and the incidence of cervical cancer [12].

The above dire global statistics has resulted in scaled-up efforts by the World Health Organization (WHO) to recently launching the Global Strategy to Accelerate the Elimination of cervical cancer, outlining three key steps including vaccination, screening and treatment. This three-pronged strategy is expected to reduce more than 40% of new cases of the disease and 5 million related deaths by 2050 if successfully implemented [1, 10]. Therefore, effective and comprehensive primary (HPV vaccination) and secondary prevention approaches (screening and treatment) are imperative [6]. Cervical cancer screening aims to early detect precancerous cervical lesions in healthy women before developing into cancer [8, 13]. Early detection and appropriate treatment are possible if robust screening is implemented [8]. Therefore, the WHO has set a target to get at least 70% of women globally screened using a high-performance test such (i.e., HPV test) by age 35 years and again by the age of 45 years by 2030 [10, 14]. However, in resource-constrained setting like Cameroon, screening is commonly done by visual methods, i.e., visual inspection of the cervix with acetic acid (VIA) or Lugol’s Iodine (VILI) which are considered low performance tests [15].

Evidence shows a significant decline in cervical cancer cases and its related mortalities in high-income countries, and this is largely attributed to the widespread screening programs [16]. On the contrary, the disease burden in SSA is due to a lack of accessible screening and treatment options, health system barriers, low levels of awareness, and challenges with health-seeking behaviours [16–18]. This reiterates the need for targeted efforts to increase the uptake of cervical cancer screening in SSA [16].

Cameroon, an SSA country, has about half (50.1%) of its population being females and at risk of the disease [19]. Nationally, the disease is the second most common cancer in women and the leading cause of cancer-related deaths [19], with a reported prevalence of 19.6 and 24% among childbearing women in two different districts [20, 21]. It ranks second in the annual cancer cases, with over 15,700 new cases annually and a mortality-to-incidence ratio above 65% [22]. The country has in the past, initiated the Cameroon National Committee for the Fight against Cancers, National Cancer Control Plan for 2003–2007 and 2006–2010, and the National Strategic Plan for Cervical Cancer Control 2015–2020 [21, 23]. Notwithstanding, the country does not have a national screening programme but rather relies on sporadic cervical cancer screenings organised by civil society organisations, and non-governmental organisations [24]. Evidence shows that one-third of these cervical cancer cases can be controlled by early detection through screening and prompt treatment [23]. Although a study conducted in the Kumbo West Health District of Cameroon reported an encouraging cervical cancer screening uptake in Cameroon to be 19.6% [20], this is not a reflection of the national reality.

Beyond the aforementioned structural limitations to cervical cancer screenings, there are some individual and contextual factors that potentially affect individuals’ decision to the screening uptakes. Other studies conducted in Cameroon have also reported multiple, individual sociodemographic factors such as age, level of education, place of residence, and marital status to have some association with the uptake of cervical cancer screenings [21, 24, 25]. However, these studies were not nationally representative as they reflected only the situation in Buea, the South-West Region of Cameroon. Therefore, a gap exists in the national prevalence of cervical cancer screenings and their associated correlates. Hence, the current study examined the prevalence and correlates of cervical cancer screening among Cameroonian women using data from the 2018 Demographic and Health Survey. The study is relevant for revamping efforts to increase cervical cancer screening among women, as well as contribute to the WHO triple-intervention strategy that envisions that by 2030, 70% of women will be screened with a high-performance test two times per life by 35 and 45 years of age [10, 14].

## Methods

### Data and materials

This study relied on the female dataset of the 2018 Cameroon Demographic and Health Surveys (CDHS). The CDHS was conducted through a two-stage sampling methodology [26]. During the 2018 CDHS project, an estimated 97.5% of the reproductive age women (15–49 years) responded and were interviewed [26]. Sample weights were generated to account for non-response. The two-stage sampling design comprised mainly of the identification of strata and Primary Sampling Units [26]. Each of the 11 regions of Cameroon, except Yaounde, were subdivided into urban-rural areas. These subdivisions are identified as 21 strata [26]. A total of 430 Primary Sampling Units (PSUs) were nested within the 21 strata. After applying the weight variable, the population size on which statistical estimations were made was 13,616.

### Measures

#### Outcome variable

The study sought to estimate the prevalence of cervical cancer screening and its correlates. Therefore, we selected this variable in the dataset: “ever tested for cervical cancer.” This response option to this variable was “Yes” or “No” or “Don’t know.” The “No” or “Don’t know” were grouped and coded as “0” in the dataset and the “Yes” coded as “1.”

#### Explanatory variables

The following sociodemographic factors were identified and selected as explanatory variables based on the review of the literature [7, 11] and their availability in the dataset: age, education, marital status, household wealth index, place of residence, and region of residence.

### Statistical analysis

Frequency, percentages, and cross-tabulation were used to describe the sample. The prevalence of cervical cancer screening was approximately 4% in the female population, rendering the dataset imbalanced or sparse [27–29]. Reliance on the Maximum Likelihood Estimation (MLE) based logistic regression will produce coefficients that are biased and unreliable [27–29]. In such situations, a data analyst has a choice among the exact logistic regression, penalized maximum likelihood estimation (PMLE), and the Firth logistic regression. The exact logistic regression, although ideal, is computationally intensive [27, 28]. The Firth logistic regression, although fairly new, can easily be computed in most statistical software. In 1993, David Firth introduced a statistical method that corrects the MLE bias that is named after the author as Firth logistic regression [30]. Packages exist in multiple statistical software to perform this analysis, and it has been established that the Firth logistic regression produces accurate results more consistently [27–29]. For our study, we implemented the Firth logistic regression using the “firthlogit” command in STATA to perform the bivariate analyses between the outcome variable and each of the explanatory variables. Given that all the explanatory variables were statistically significant correlates, they were all adjusted for in a multivariable analysis. All analyses were performed in STATA version 14.

### Ethical considerations

The study was performed in accordance with the Declaration of Helsinki and approved by appropriate ethics committee. Ethical clearance was obtained from the Ethical Review Committee of Ministère de la Santé Publique Yaoundé, Cameroun and the Institutional Review Board of ICF International [26]. Informed consents were obtained from participants prior to data collection. We obtained permission from the DHS program to use the 2018 CDHS for our study at https://dhsprogram.com/data/dataset_admin/index.cfm. All data were anonymized before the authors received the data. All methods were performed in accordance with the relevant guidelines and regulations.

## Results

### Sample description

Only about 4% of reproductive age women in Cameroon ever screened for cervical cancer screening (Table 1). North-West recorded the highest cervical cancer screening prevalence (9.35%) followed by Douala (7.54%). Seven out of ten have ever screened for HIV. The women were generally young (28 years ±9). Many of them have attained secondary school level education (45.2%), were currently married (56.9%) and dwelt in urban areas (55.4%). The women were in socioeconomically diverse households. Details of the sample description statistics are reported in Table 1.

**Table 1:** Weighted summary statistics of study variables (*N* = 13,616)

Covariates	Total		
	n (%)	Cervical Cancer Screening	
		No (%)	Yes (%)
Total sample	13,616 (100)	13,145 (96.54)	471 (3.46)
**HIV screening**	***p*** **≤ 0.001**		
No	3926 (28.83)	99.66	0.34
Yes	9689 (71.17)	95.28	4.72
**Age** (M = 27.80, SD = 9.44)	***p*** **≤ 0.001**		
15–24	5726 (42.06)	98.94	1.06
25–34	4398 (32.30)	96.15	3.85
35–44	2588 (19.01)	93.78	6.22
45+	903 (6.63)	91.11	8.89
**Education**	***p*** **≤ 0.001**		
None	2778 (20.41)	99.27	0.73
Primary	3630 (26.66)	97.09	2.91
Secondary	6158 (45.23)	96.01	3.99
Higher	1049 (7.70)	90.52	9.48
**Marital Status**	***p*** **≤ 0.001**		
Never	4692 (34.46)	98.11	1.89
Currently married	7748 (56.91)	95.82	4.18
Previously married	1175 (8.63)	95.01	4.99
**Household wealth**	***p*** **≤ 0.001**		
Poorest	2239 (16.44)	99.59	0.41
Poorer	2502 (18.37)	98.28	1.72
Middle	2696 (19.80)	97.77	2.23
Richer	2939 (21.58)	96.55	3.45
Richest	3241 (23.80)	92.05	7.95
**Rural-Urban residence**	***p*** **≤ 0.001**		
Urban	7538 (55.36)	94.91	5.09
Rural	6078 (44.64)	98.56	1.44
**Region of residence**	***p*** **≤ 0.001**		
Adamawa	630 (4.62)	98.90	1.10
Centre (without Yaounde)	1350 (9.91)	98.24	1.76
Douala	1675 (12.30)	92.46	7.54
East	848 (6.23)	96.96	3.04
Far-North	2009 (14.76)	99.95	0.05
Littoral (without Douala)	507 (3.72)	96.72	3.28
North	1720 (12.64)	99.51	0.49
North-West	882 (6.48)	90.65	9.35
West	1442 (10.59)	95.46	4.54
South	723 (5.31)	98.36	1.64
South-West	306 (2.25)	94.56	5.44
Yaounde	1522 (11.18)	94.38	5.62

### Correlates of cervical cancer screening in Cameroon

Firth logistic regression was used to assess the relationship between sociodemographic variables in both bivariate and multivariable models. The study variables were all statistically significantly related to the outcome variable (Table 2). In the adjusted model, the results indicated that women with the following sociodemographic characteristics have a higher likelihood of undergoing cervical cancer screening: ever undergone HIV screening (AOR = 4.446, 95% CI: 2.475, 7.986), being 24–34 years (AOR = 2.233, 95% CI: 1.606, 3.103) or 35–44 years (AOR = 4.008, 95% CI: 2.840, 5.657) or having at least 45 years (AOR = 5.895, 95% CI: 3.957, 8.784), having attained a post-secondary education (AOR = 1.849, 95% CI: 1.032, 3.315), currently (AOR = 1.551, 95% CI: 1.177, 2.043) or previously married (AOR = 1.572, 95% CI: 1.073, 2.302), dwelling in the richest household (AOR = 4.139, 95% CI: 1.769, 9.682), residing in an urban area (AOR = 1.403, 95% CI: 1.004,1.960), and residing in North-West (AOR = 3.435, 95% CI: 2.403, 4.910). Conversely, women who resided in Adamawa (AOR = 0.436, 95% CI: 0.213, 0.892) or Centre (without Yaounde) (AOR = 0.538, 95% CI: 0.333, 0.871) or Far-North (AOR = 0.063, 95% CI: 0.012, 0.324) or North (AOR = 0.237, 95% CI: 0.107, 0.526) or South (AOR = 0.340, 95% CI: 0.197, 0.587) had a lower likelihood of undergoing cervical cancer screening. See Table 2 for details.

**Table 2:** Crude and adjusted firth logit models showing the correlates of cervical cancer screening (*N* = 13,616)

	OR [95% CI]	AOR [95% CI]
**HIV screening**		
No	Ref.	Ref.
Yes	14.40*** [8.198,25.30]	4.446*** [2.475,7.986]
**Age**		
15–24	Ref.	Ref.
25–34	3.712*** [2.760,4.993]	2.233*** [1.606,3.103]
35–44	6.006*** [4.453,8.099]	4.008*** [2.840,5.657]
45+	8.207*** [5.805,11.60]	5.895*** [3.957,8.784]
**Education**		
None	Ref.	Ref.
Primary	3.345*** [2.056,5.444]	1.006 [0.589,1.718]
Secondary	4.635*** [2.914,7.372]	1.284 [0.753,2.190]
Higher	12.58*** [7.672,20.62]	1.849* [1.032,3.315]
**Marital Status**		
Never	Ref.	Ref.
Currently married	2.122*** [1.678,2.685]	1.551** [1.177,2.043]
Previously married	2.637*** [1.893,3.673]	1.572* [1.073,2.302]
**Household wealth**		
Poorest	Ref.	Ref.
Poorer	4.319*** [1.990,9.373]	1.771 [0.788,3.983]
Middle	5.076*** [2.378,10.84]	1.714 [0.756,3.887]
Richer	8.714*** [4.142,18.33]	2.229 [0.961,5.168]
Richest	20.37*** [9.823,42.26]	4.139** [1.769,9.682]
**Rural-Urban residence**		
Rural	Ref.	Ref.
Urban	3.526*** [2.791,4.455]	1.403* [1.004,1.960]
**Region of residence**		
Yaounde	Ref.	Ref.
Adamawa	0.138*** [0.0704,0.272]	0.436* [0.213,0.892]
Centre (without Yaounde)	0.262*** [0.167,0.411]	0.538* [0.333,0.871]
Douala	1.159 [0.851,1.579]	1.048 [0.762,1.443]
East	0.398*** [0.262,0.604]	0.875 [0.560,1.366]
Far-North	0.0152*** [0.00302,0.0764]	0.0625*** [0.0121,0.324]
Littoral (without Douala)	0.548** [0.361,0.833]	0.853 [0.549,1.325]
North	0.0777*** [0.0367,0.165]	0.237*** [0.107,0.526]
North-West	1.805*** [1.314,2.481]	3.435*** [2.403,4.910]
West	0.593** [0.417,0.845]	1.078 [0.733,1.585]
South	0.178*** [0.105,0.299]	0.340*** [0.197,0.587]
South-West	0.944 [0.589,1.513]	0.994 [0.612,1.616]

Exponentiated coefficients; 95% confidence intervals in brackets

* *p* < 0.05, ** *p* < 0.01, *** *p* < 0.001

## Discussion

Cervical cancer screening in Cameroon remains low despite the high mortality rate of cervical cancers cases. This study analyzed the Cameroon 2018 Demographic and Health Surveys datasets. Currently, Cameroon does not have a national cervical cancer screening program. Factors such as HIV screening, age, education, marital status, and household wealth were found to be associated with cervical cancer screening using crude and adjusted firth logit modelling. Findings from this study suggest that as low as 3.5% of women of reproductive ages in Cameroon have ever been screened for cervical cancer. This is low when compared to the screening rate reported in other studies. Studies done in other SSA countries have also reported a low uptake in cervical cancer screening: 21% in Tanzania [31], 3% in Ghana [32], 4.8% in Uganda [33], and 6% in Kenya [34]. Cervical cancer screening undoubtedly leads to a reduction in cervical cancer burden as evidenced by reports from some developed countries [32]. However, in the least developed and developing countries in SSA, the low number of screening programs have led to increased morbidity and mortality from cervical cancers due to lack of early detection [35–37]. According to the WHO, cervical cancer screening leads to early detection of both precancerous and cervical lesions preventing serious disease and improving prognosis [38]. This signifies the need for the institution of cervical cancer screening programs in Cameroon.

The odds of screening for cervical cancer significantly increased with increasing age and level of education. As expected, women aged 45 years and above were 8 times as likely to be screened for cervical cancer compared to women of younger age. This result is consistent with a study done in South Africa which found that women who are 30 years and above were more likely to utilize cervical cancer screening compared to younger women [39]. Also, a study done by Muluken and colleagues (2020) in Ethiopia revealed that older women were more receptive to cervical cancer screening compared to their younger counterparts [40]. However, a study done in Uganda found a decreased odd in cervical cancer screening with increasing age [33]. The average age of cervical cancer diagnosis is 50 years with most women diagnosed between the ages of 35 and 44 years, globally [41]. This may explain the reason for increased uptake of cervical cancer screening with increasing age. Besides, higher education significantly increased the odds of cervical cancer screening in this study. This concurs with the findings of two studies done in Ghana and Ethiopia that showed that cervical cancer screening utilization decreased with a lack of formal education [42, 43]. Awareness of cervical cancer screening should be intensified in Cameroon targeted towards young women and the less educated.

In our study, women who underwent HIV screening were more likely to screen for cervical cancer. The risk of HPV infection leading to cervical abnormalities is very high in women with HIV infection [44]; therefore, women who tested for HIV and knew their status will have a higher likelihood to test for cancers. Studies conducted in Botswana [45], Western Kenya [46], and Ethiopia [47] found a significant increase in the utilization of cervical cancer screening services with HIV positivity. In addition, this study revealed that married women were more likely to utilize cervical cancer screening services compared to women who have never been married. Though some studies have found increased uptake of cervical cancer screening among married women compared to unmarried women [13, 48], the reverse is also established in other studies [32, 40]. These point to the mixed effect of marriage on the uptake of cervical cancer screening. The use of different classifications of marital status by different studies and the unique cultural values that exist in various countries in Africa may be the reasons for this observation.

We found that cervical cancer uptake increases with an increase in income and urbanity. Though Cameroon health insurance ensures free-provision of some health services, most services are based on itemized fee-for-service. In the past decade, Cameroon has spent more money on healthcare per capita ($61.00) than any other SSA country apart from South Africa, but the distribution of health services has striking geographic disparities [49]. Women who dwelt in urban areas generally have easy access to health services, compared to rural dwellers. Residing in a rural area is a risk factor because cervical cancer screening in Cameroon is done by a few public hospitals that are mainly located in urban areas. Except for the North-West region, residing in some five regions, compared to Yaounde, was negatively associated with cervical cancer screening. Though the Centre Region, including the capital (Yaoundé), contains only 18% of the population, 40% of the country’s medical doctors practice there; conversely, 8% of the nation’s medical doctors, practice in the Far North which has approximately the same population size as the Centre Region [49]. Women who dwelt in regions outside the capital were associated with lower odds of screening for cervical cancer, this might be due to limited and inequitable access to health resources and facilities. High cervical cancer screening prevalence in North-West can mainly be attributed to a successful implementation of a nurse-led cervical cancer screening program using visual inspection with acetic acid enhanced by digital cervicography (VIA-DC) [50]. The VIA-DC program was part of the Women’s Health Program (WHP) founded by the Cameroon Baptist Convention Health Services (CBCHS) [50]. The VIA-DC’s program started in 2007, mainly in the western regions (North-West, South-West, and West) [50]. For North-West, the VIA-DC implementation covered large catchment areas compared to the other western regions [50]. The VIA-DC program was implemented through the Banso and Mbingo Baptist hospitals and Nkwen Baptist integrated health centre [50].

The study has strengths and limitations that are worthy of mention. The Cameroon Demographic Health and Survey datasets are large and nationally representative. We employed Firth-logit modelling to handle the sparsity of the dataset. Also, the data supporting this study are nearly nationally representative suggesting that our results are generalizable for the entire country except for some zones in rural areas of South-West region. Data was not collected from these zones because enumerators were not permitted into the area due to security concerns [51]. Although data from the South-West region may be representative of its urban areas, the estimated prevalence for the region should be interpreted with this limitation in mind. We, however, do not expect that data on cervical cancer screening from the exempted zones can make a big impact on the national estimate of cervical cancer screening prevalence. Another important strength is that we have estimated the prevalence and unravelled the correlates of cervical cancer screening in Cameroon which is key for policymaking and implementation. However, a key limitation of our study is that the study used secondary data which utilized a cross-sectional design. Hence, the associations observed in this study do not infer a causal relationship between the correlates and the outcome variables. The study was also restricted to variables available in the Cameroon Demographic and Health Survey datasets.

Based on our findings we recommend that the Cameroon National Committee for Fight Against Cancer be empowered to achieve its objectives of drafting cancer control policies and strategies, cancer prevention, early diagnosis of cancer, and cancer research. Though in 2015 Cameroon completed the pilot phase of rolling out HPV vaccines for girls aged 9–13 years, the Ministry of Health has not been able to add it to its routine expanded program on immunization [EPI]. We recommend the introduction of HPV vaccination into routine EPI as it is one of the most effective ways of reducing the incidence of cervical cancer.

At the moment, most cervical cancer screening services are done by some public hospitals in some cities and two (2) denominational structures in Cameroon namely Baptist Convention Health Services and the Presbyterian Church in Cameroon. There is, however, a need for the establishment of a national screening program for cervical cancers that will serve both urban and rural areas. The national program should also address barriers to cervical cancer screening uptake to ensure smooth implementation of the interventions. There is also a need for sensitization, through community and media campaigns especially in rural areas by community health workers on the need for cervical cancer screening.

## Conclusions

Generally, the proportion of Cameroonian women who have ever screened for cervical cancer continue to remain low at about 4%. In the adjusted multivariate model, women with increasing age (45 years and above), had higher education, were married, were HIV positive, lived in urban areas and had increasing household wealth were more likely to screen for cervical cancer. Therefore, policy interventions should target Cameroonian women who are younger, less educated, land those who dwell in poor households and rural areas. A national screening program for cervical cancers that serves both urban and rural residents should be established.

## Data Availability

Data for this study were sourced from Demographic and Health surveys (DHS) and available here: http://dhsprogram.com/data/available-datasets.cfm.

## Abbreviations

CDHS: Cameroon Demographic and Health Survey
EPI: Expanded Program on Immunization
HPV: Human Papillomavirus
HIV: Human Immunodeficiency Virus
LMICs: Low-and-middle-income countries
MLE: Maximum Likelihood Estimation
PMLE: Penalized Maximum Likelihood Estimation
PSUs: Primary Sampling Units
SSA: Sub-Saharan Africa
WHO: World Health Organisation
